# Live fast, die fast principle in a single cell of fission yeast

**DOI:** 10.15698/mic2017.09.591

**Published:** 2017-08-13

**Authors:** Hidenori Nakaoka

**Affiliations:** 1 Department of Basic Science, Graduate School of Arts and Sciences, The University of Tokyo.

**Keywords:** macroscopic phenomenology, growth, death, aging, protein aggregation, microfluidics, fission yeast

## Abstract

Growth and death are both fundamental macroscopic properties for all living matters, and thus cell division and mortality rates are good parameters for characterizing cellular physiology in a given environment. While population growth rates in various conditions have been reported in literature, death rate is rarely measured, especially in favorable culture conditions where cells grow exponentially. In our recent study (Nakaoka and Wakamoto, 2017), we developed a microfluidics-based platform to track multiple single cell lineages until death. The system enabled us to monitor both cell growth and death in controlled steady environments, and we confirmed the absence of replicative aging in fission yeast old-pole cell lineages by showing remarkable constancy both in cell division and mortality rates. Furthermore, we revealed a growth-death trade-off relation in non-stressed conditions. The phenomenological law that constrains macroscopic physiological parameters could provide a new quantitative insight into possible balanced-growth states in various environments.

## Fission yeast old-pole cell lineages do not age under favorable conditions

Replicative senescence or aging in unicellular organisms is defined as progressive decline in proliferative activity and increase in mortality rate as cells undergo divisions. In budding yeast *Saccharomyces cerevisiae*, which is one of the most popular model microorganisms, a mother cell typically produces 20-30 daughter cells before death. The mother cell undergoes replicative aging, and molecular markers correlated with the aging phenotypes, such as ERCs (extra-chromosomal rDNA circles), high cytosolic and vacuolar pH, carbonylated proteins (oxidative damages on proteins), and protein aggregation, have been postulated. Substantial evidence suggests that those “aging factors” are preferentially retained in mother cell lineages by several mechanisms including septin-dependent lateral diffusion barriers at bud necks, confinement on organelles, and actin-cable dependent retrograde flow. Because mother cells are larger than their daughters in general, it is easy to identify and track aging lineages, which makes budding yeast a favored model organism in aging studies.

**Figure 1:**
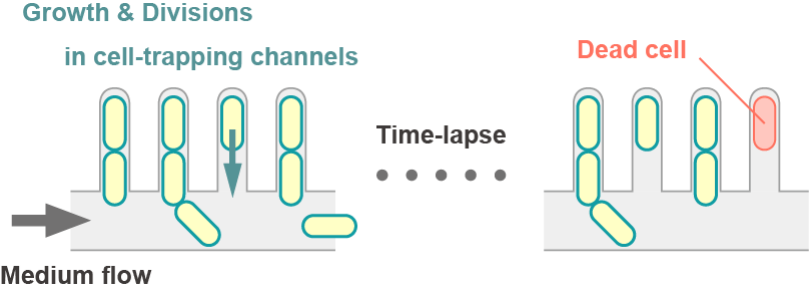
FIGURE 1: Microfluidics for tracking individual cell lineages. Fission yeast cells are trapped in comb-like thin micro-channels. Fresh medium is constantly supplied through a thick trench to make the external environment unchanged during measurements. Daughters produced by the trapped cells (= old-pole cells) at the end of the channels are washed away by the medium flow, preventing the cell population in the device from saturation. Automated time-lapse microscopy allows us to simultaneously track a large number of independent old-pole cell lineages until death.

Another well-studied model yeast is the fission yeast *Schizosaccharomyces pombe*, which divides by binary fission. In contrast to budding yeast, two siblings upon cell divisions are morphologically indistinguishable. Although any forms of “damages” can be asymmetrically partitioned even in the symmetrically dividing yeast, no molecular mechanisms that actively specify aging lineages have been suggested, and thus a “random damage segregation” model is preferred. Indeed, recent works including ours have suggested the absence of replicative aging in old-pole cell lineages in *S. pombe*. We developed a microfluidic device that can track 1,000-2,000 independent old-pole cell lineages in steady environments (Figure 1), and showed that both cell division and death rates are remarkably constant for at least ~100 generations. We further demonstrated that Hsp104-associated protein aggregates tended to remain at old poles, but can sometimes move toward new pole ends, freeing the old-pole cells from the aggregation load. The segregation of the protein aggregates to the new poles were random events with a characteristic time scale of ~10 generations, supporting the idea that asymmetrically distributed cellular materials are not strictly confined within specific lineages but randomly partitioned to one of the two siblings in fission yeast.

The lack of senescence in the old-pole lineages in fission yeast is not only biologically intriguing but also technically important because it can provide a convenient experimental set-up where one does not need to consider age-dependency in any biological phenomena of interest, making interpretations clear and easy.

## Stochastic cell death in favorable culture conditions

Microbiologists routinely culture their favorite model microbes in “appropriate” conditions: well-established rich and/or chemically defined media and optimum temperatures. Those culture conditions support fast exponential growth of the cells. But to what extent are the cells really happy with those environments? More specifically, what are the cell death rates in the “favorable” conditions? This simple question has been difficult to answer because rare death events are hardly observed in the sea of exponentially growing cells in conventional batch cultures. The microfluidic system enabled us to track each single cell lineage as long as we want, that is, until cell death. With this technical break-through, we measured cellular division and death frequencies in 7 different environments that are within the range of standard culture conditions. Our work demonstrated that fission yeast old-pole cell lineages are aging free (or at least the aging processes are extremely slow compared to other organisms) in all those non-stressed conditions, but at the same time they are not immortal with a death probability of 0.5-1.2% per generation.

Interestingly, we observed that Hsp104-associated protein aggregation was abruptly and significantly accelerated just before death, suggesting that the cell death was associated with acute physiological alterations. The observation is consistent with previously reported correlation between protein aggregation and cell death. Importantly, however, our data showed that the sudden acceleration in aggregation can take place regardless of whether the cell contained large amount of protein aggregates or not, suggesting that protein aggregates *per se* are not triggering the onset of the death process. Future work will identify the primary cues other than protein aggregation that cause the stochastic catastrophe in cells. Are specific molecular signaling cascades responsible for the death process for all the cases, or can they be different between cells? By addressing those questions, we might be able to understand how cellular homeostasis can be broken even without explicit sources of external stress.

## Trade-off between growth and death

**Figure 2:**
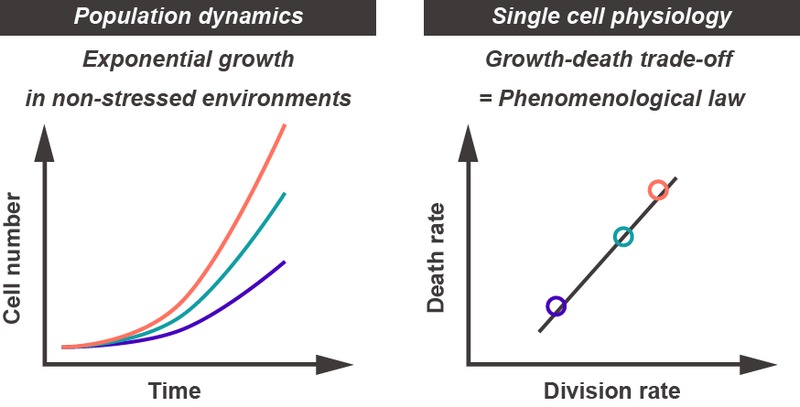
FIGURE 2: The growth-death trade-off in favorable conditions. Populations of cells exhibit exponential growth in favorable culture conditions (left). However, we cannot tell specific death rates in the environments from the growth curves. The lineage tracking approach enabled us to directly measure division and death rates that are inherent to single cells, and revealed the hidden trade-off relation between growth and death (right). This is an example for a macroscopic phenomenological law that governs cellular physiological states.

Our results revealed that there is a specific death rate in a given non-stressed environment, leading to the question how death and growth rates are related to each other. We found that there is a positive, almost linear correlation between the two rates, implying that not all aspects of physiology of the cells in the faster growing population are better than those in slower growing populations. The population fitness gain is, in fact, achieved at the cost of shorter expected life spans of the constituent individual cells. This can be thought as one of the biological principles at the single cell level, which is hidden under the population level dynamics (Figure 2). Quantitative measurements at the single cell level have become a growing trend in modern biology, and will be more and more important in the future.

What else can we learn from the growth-death trade-off? The relation might quantitatively define “steady and balanced growth states”, where every cellular component doubles in amount in each division. Deviations from the trend might be indicative of non-steady state growth of the population. Indeed, we found that in the recovery phase from transient oxidative stress treatment, growth rate quickly resumed while death rate slowly returned to normal; imbalance between growth and death rates can be realized, but it is a transient relationship before the cell population returned to the steady state in the given environment. In a broader context, we think that the constraint on growth and death rates might be one of the macroscopic phenomenological laws in biology. Because a cell is a complex system comprised of a whole bunch of biomolecules, it resides in a high dimensional space in principle, hampering our intuitive understanding of cellular state and its dynamics. Focusing on macroscopic physiological parameters, such as growth and death rates, would be an alternative and complementary approach to studies at the molecular level. Our work presents an interesting example of characterizing global cellular states with a small number of parameters.

Finally, we would like to briefly discuss possible extensions for the growth-death relations. Because our current work is limited to non-stressed culture conditions, the trade-off relation does not represent the entire picture of the growth-death balances. Rather, there is a good possibility that the simple trade-off does not apply for stressed conditions. Systematic measurements for various environments including stress conditions will be the next challenge for revealing the ranges of the possible growth-death balances. Also, the universality for the relation is not still clear. Other organisms and different fission yeast strains should be examined. For example, there are several wild-type *S. pombe* strains used in labs, and very recent work has shown that death rates in a rich environment are different among those strains. It would be interesting to ask if growth and death are linearly correlated in them too, and if so, the quantitative comparison between the slopes or X-intercepts of the lines might provide insights into the origin of the growth-death balances, and the mechanism of homeostatic regulation in cellular systems.

